# Efficacy and safety of fostamatinib in refractory immune thrombocytopenia: a meta-analysis from randomized controlled trials

**DOI:** 10.1007/s00277-024-05824-7

**Published:** 2024-06-10

**Authors:** Songphol Tungjitviboonkun, Naharuthai Bumrungratanayos, Jedsadakorn Jitwimungsanon, Thanat Kheamakulvanich, Smuch Siramongkholkarn

**Affiliations:** 1 University of California San Francisco, San Francisco, United States; 2 Division of Hematology, Department of Medicine, Sirindhorn Hospital, Bangkok, Thailand; 3 HRH Princess Maha Chakri Sirindhorn Medical Center, Faculty of Medicine, Srinakharinwirot University, Nakhon Nayok, Thailand; 4 Koh Sichang Hospital, Chonburi, Thailand; 5 Department of Medicine, Faculty of Medicine Ramathibodi Hospital, Mahidol University, Bangkok, Thailand

**Keywords:** Fostamatinib, Spleen tyrosine kinase, SYK inhibitor, Immune thrombocytopenia, ITP, Refractory ITP, Meta-analysis

## Abstract

**Background:**

Immune thrombocytopenia (ITP) is an immune-mediated disease that results in low platelet counts. Despite appropriate treatment, many patients continue to experience refractory disease. Fostamatinib, an oral spleen tyrosine kinase (SYK) inhibitor, has emerged as a promising option for refractory ITP.

**Objective:**

This meta-analysis aims to evaluate the efficacy and safety of fostamatinib compared to conventional therapy in adults aged ≥ 18 years with refractory ITP.

**Materials and methods:**

Literature search was conducted in PubMed, Scopus, Embase, and clinicaltrials.gov databases from inception to March 31, 2024. Randomized controlled trials (RCTs) assessing the safety and efficacy of fostamatinib in adults with refractory ITP were included. Data extraction, risk of bias assessment, and statistical analysis were performed following PRISMA guideline.

**Results:**

A total of 495 articles were screened, with three RCTs meeting the inclusion criteria. Fostamatinib therapy demonstrated superior efficacy in achieving stable platelet response by week 24 (ORR 0.80; 95%CI 0.72–0.88), platelet count ≥ 50,000/µL at weeks 12 (ORR 0.80; 95%CI 0.72–0.90) and week 24 (ORR 0.82; 95%CI 0.72–0.90). Additionally, fostamatinib improves platelet counts in subjects with a baseline count of < 15,000/µL. The Number Needed to Treat (NNT) was calculated as 10. Adverse effects include diarrhea (RR 2.32; 95%CI 1.11–4.84), hypertension (RR 2.33; 95%CI 1.00-5.43), and abnormal liver function tests (RR 4.18; 95% CI 1.00-17.48). Interestingly, the occurrences of nausea (RR 1.77; 95% CI 0.33–9.67) and rash (RR 2.28; 95% CI 0.50-10.29) did not achieve statistical significance.

**Conclusion:**

This meta-analysis provides robust evidence supporting the efficacy of fostamatinib in improving platelet counts and achieving therapeutic goals in adults with refractory ITP. However, fostamatinib’s safety profile warrants consideration due to higher rates of diarrhea, hypertension, and abnormal liver function tests.

**Supplementary Information:**

The online version contains supplementary material available at 10.1007/s00277-024-05824-7.

## Introduction

Immune thrombocytopenia (ITP) is an autoimmune disorder characterized by a low platelet count, which can lead to an increased risk of bleeding and other complications [1]. The incidence of ITP was reported to be 3.3 adults per 100,000 per year [2]. Corticosteroids serve as the first-line treatment for ITP [3]. Despite the availability of various treatment options, a significant proportion of adults with ITP experience refractory disease, wherein conventional therapies either fail to provide an adequate platelet response or are associated with intolerable side effects.

Spleen tyrosine kinase (SYK) is expressed primarily in hematopoietic cells and plays a key role in the signaling of activating Fc receptor (FcR) and the B-cell receptor (BCR) [4]. Fostamatinib (formerly R788), an oral SYK inhibitor, was initially developed for rheumatoid arthritis treatment [5, 6]. By inhibiting phosphorylation of SYK substrate linker for activation of T cell and B cell, fostamatinib reduces antibody-mediated and immune-complex-mediated inflammation [7, 8]. Fostamatinib later received FDA approval in April 2018 as a promising therapeutic option for patients with refractory ITP [9]. In patients with ITP, the fostamatinib dose started at 100 mg twice daily and can be increased up to 150 mg twice daily [8]. Previous randomized controlled trials (RCTs) have assessed the efficacy and safety of fostamatinib compared to other treatments in this patient population [10, 11]. However, the results of these studies have shown variability, and adverse effects have not been statistically described. Thus, there is a need for a comprehensive synthesis of existing evidence to determine the overall efficacy and safety profile of fostamatinib in refractory ITP.

This meta-analysis aims to systematically review and analyze the available RCTs to evaluate the efficacy and safety of fostamatinib in combination with conventional therapy compared to conventional therapy alone in adults aged ≥ 18 years with refractory ITP. The primary outcomes of interest include stable platelet response by week 24, platelet count at week 12 and week 24, and achievement of platelet count goals in subjects with a baseline count < 15,000/µL.

By synthesizing the existing evidence, this meta-analysis aims to provide clinicians, researchers, and policymakers with valuable insights into the potential benefits and risks of fostamatinib in the management of refractory ITP, ultimately informing clinical decision-making and guiding future research efforts in this area.

## Materials and methods

### Objective

The aim of this meta-analysis was to evaluate the efficacy and safety of fostamatinib in combination with conventional therapy compared to conventional therapy alone in adults aged ≥ 18 years with refractory immune thrombocytopenia (ITP). The primary outcome in this study is efficacy measures by using the Objective Response Rate (ORR). The ORR was assessed based on stable platelet response by week 24, platelet count ≥ 50,000/µL at weeks 12 and 24, and achievement of a count ≥ 30,000/µL and at least 20,000/µL above baseline at weeks 12 and 24 in subjects with a baseline count < 15,000/µL. The secondary outcome measures focused on adverse events associated with fostamatinib treatment.

### Search strategy

A comprehensive literature search was conducted using the following databases: PubMed (MEDLINE), Scopus, Embase, and the Registry of clinicaltrials.gov. The search strategy employed the keywords “Fostamatinib and Immune thrombocytopenia” or “Fostamatinib and ITP”. The literature search encompassed articles published from the inception of each database up to March 31, 2024. The search was restricted to articles published in English only. Search queries for each database can be found in the supplementary file. The authors adhered to PRISMA 2020 guideline [12]. 

### Inclusion and exclusion criteria

Inclusion criteria: Studies that provided data on the safety and efficacy of fostamatinib in adults with refractory ITP.

Exclusion criteria: Review articles, case reports, case series, preclinical studies, and clinical studies irrelevant to fostamatinib or ITP. Studies without safety or efficacy outcomes were also excluded.

### Study selection

Two authors (J.J. and T.K.) independently screened the titles and abstracts of the identified articles. Any discrepancies in the screening process were resolved by a third researcher (S.T.).

### Data extraction

Data extraction was performed independently by two authors (N.B. and S.T.) using a standardized data extraction form. The extracted data included baseline characteristics, efficacy outcomes (stable platelet response by week 24, platelet count at week 12 and week 24, achievement of platelet count goals in subjects with baseline count < 15,000/µL), and adverse events. In instances where data were missing or unclear, we made assumptions based on available information and consulted with other experts in the field. The full data extraction form can be found in the supplementary file.

### Risk of bias assessment

The risk of bias in the included studies was assessed using the Cochrane Risk of Bias Tool (ROB-II) [13] by two independent researchers (J.J. and S.T.). Any disagreements in the assessment were resolved through discussion or consultation with a third researcher (N.B.).

### Statistical analysis

Data analysis was conducted using STATA version 18.0 software (StataCorp, College Station, TX, USA). The meta-analysis was performed to calculate pooled estimates of risk ratio, risk difference, and Number Needed to Treat (NNT) concerning both efficacy and adverse events associated with fostamatinib treatment. Heterogeneity analysis was studied, and a fixed-effects model was utilized.

## Result

**Fig. 1:**
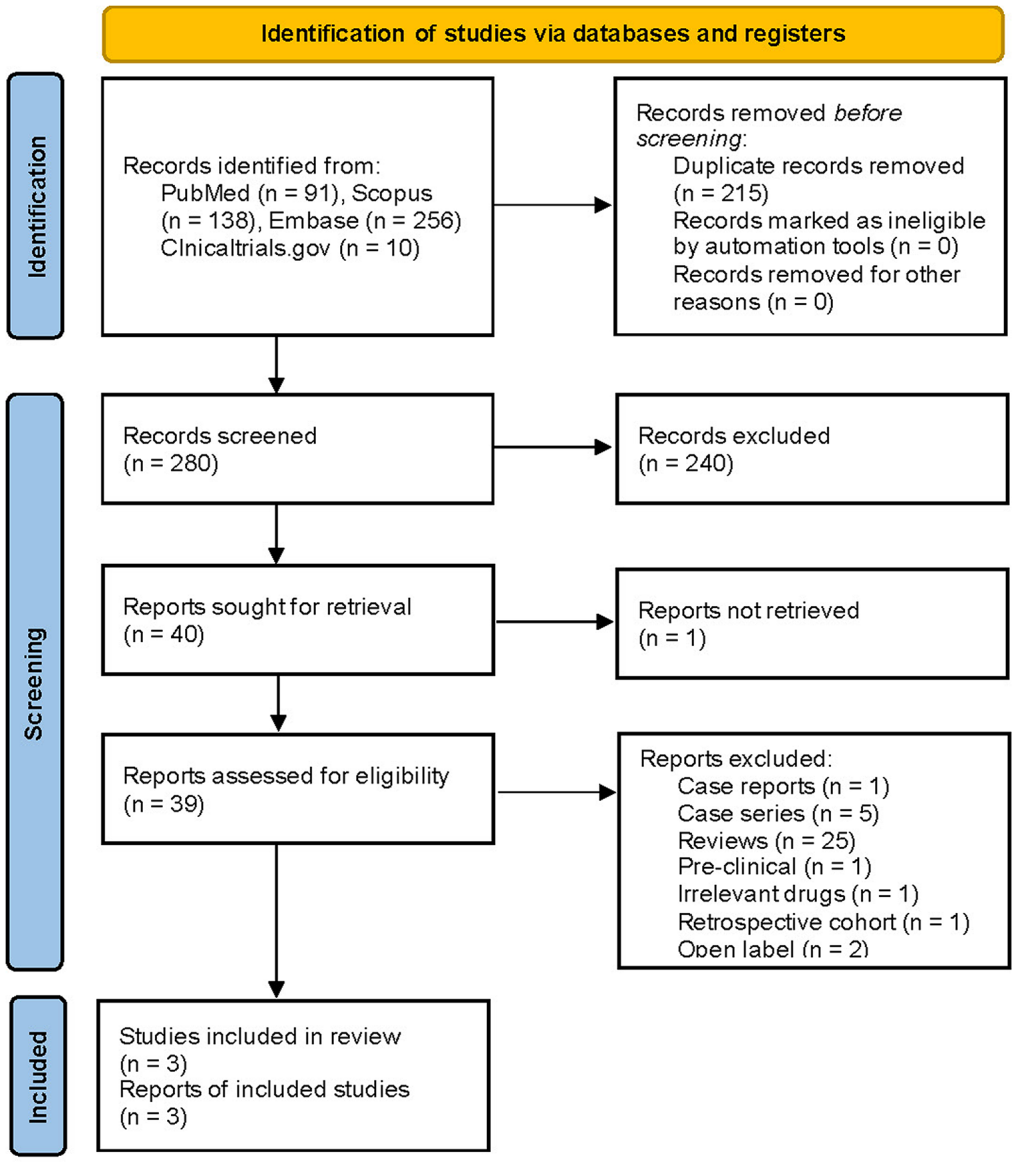
PRISMA flowsheet of selection of articles

From the database, a total of 495 articles were identified: 91 from PubMed, 138 from Scopus, 256 from Embase, and 10 from clinicaltrials.gov. After careful screening of the articles, three randomized controlled trials (*N* = 123) were included (Fig. 1).

The risk of bias was low in all studies. All studies provided detailed information about the allocation sequences, which were stratified random. Two studies balanced patients with prior splenectomy and the degree of thrombocytopenia, while one study stratified by baseline platelet count (< or ≥ 15,000/µL). Conflict arose in the allocation concealment part; while one author (J.J) rated all studies as “No information,” the other authors (S.T. and N.B) pointed out that in the context of a large trial run by an experienced clinical trials unit, responding with “Probably yes” rather than “No information” is more reasonable [13]. Our judgment differed from a previous meta-analysis, which rated it as some concern due to no information [14, 15]. No baseline differences between treatment arms were observed. The comprehensive risk of bias assessment is summarized (Supplementary Fig. 1).

### Baseline characteristics

The main characteristics of patients included in the studies are shown (Table 1). In total, these studies encompassed 184 patients with refractory ITP. Of these participants, 123 were administered fostamatinib in conjunction with conventional therapy, while 61 patients received conventional therapy alone. The dose of fostamatinib varied across the studies, with patients receiving either 100 mg PO bid per day or 150 mg PO bid per day. All patients enrolled in the studies had previously undergone 2 to 3 lines of therapy for ITP before participating in the trials. The majority of these prior treatments consisted of corticosteroids, which were utilized by over 90% of the patients. Additionally, more than 60% of the patients had previously been treated with thrombopoietin agents or immunosuppressive drugs. Upon comparing the baseline characteristics between the fostamatinib group and the conventional therapy group, no significant differences were observed. The demographic details of the patients, such as age and gender distribution were well-balanced between the two treatment groups.

### Conventional therapy

In the three randomized controlled trials (RCTs) included in our meta-analysis, the conventional therapies utilized in these studies were notably similar, contributing to low heterogeneity between the studies.

Two of the studies primarily consisted of a single medication such as corticosteroids at a dosage of < 20 mg prednisolone equivalent per day, azathioprine, or danazol throughout the study duration. Similarly, the third study permitted the use of corticosteroids at a dosage of ≤ 10 mg prednisolone per day, azathioprine, or danazol throughout the study period.

The uniformity in the conventional therapy regimens across the three studies is notable. This similarity minimizes the potential for heterogeneity in treatment effects, thereby enhancing the comparability of the study outcomes. By employing consistent treatment protocols, the studies aimed to provide a standardized control group against which the efficacy and safety of fostamatinib could be evaluated.

The adequacy of the conventional therapy dosage in the control arm of the included studies depends on several factors, including the severity of the patient’s condition, previous treatment responses, and potential side effects. While the specified corticosteroid dosage (< 20 mg prednisolone equivalent per day) is within the range commonly used in clinical practice for ITP management, the dosage threshold for azathioprine and danazol remains unspecified.

**Table 1:** Baseline characteristic of patients in included studies

Studies	Intervention/Control	Dose	*N*	M: F	Median Age	Median Baseline platelet (range)	Prior splenectomy	Prior therapy (%)
Bussel (2019) NCT02076399	Fostamatinib + one conventional therapy	100 mg PO bid or 150 mg PO bid	51	21:30	57 (20–88)	16,202 (1,000–51,000)	20 (39%)	Corticosteroids 46 (90), IVIg, or IV Anti-D 33 (65); Thrombopoietic Agents 27 (53); Immunosuppressants 22 (43)
	Conventional therapy		25	8:17	57 (26–77)	15,844 (1,000–48,000)	10 (40%)	Corticosteroids 25 (100), IVIg, or IV Anti-D 17 (68); Thrombopoietic Agents 15 (60); Immunosuppressants 12 (48)
Bussel (2019) NCT02076412	Fostamatinib + one conventional therapy	100 mg PO bid or 150 mg PO bid	50	19:31	50 (21–82)	15,900 (1,000–33,000)	14 (28%)	Corticosteroids 48 (96), IVIg, or IV Anti-D 19 (38); Thrombopoietic Agents 20 (40); Immunosuppressants 22 (44)
	Conventional therapy		24	11:13	50 (20–78)	23,958 (1,000–156,000)	9 (38%)	Corticosteroids 22 (92), IVIg, or IV Anti-D 10 (42); Thrombopoietic Agents 10 (42); Immunosuppressants 10 (42)
Kuwana (2022) NCT04132050	Fostamatinib + one conventional therapy	100 mg PO bid or 150 mg PO bid	22	4:18	61 (25–81)	19,000 (3,000–28,000)	5 (23%)	Corticosteroids 21 (95), IVIg, or IV Anti-D 8 (36); Thrombopoietic Agents 11 (50); Immunosuppressants 3 (14)
	Conventional therapy		12	4:8	64 (31–76)	18,000 (1,000–27,000)	2 (17%)	Corticosteroids 12 (100), IVIg, or IV Anti-D 4 (33); Thrombopoietic Agents 7 (58); Immunosuppressants 1 (8)

### Primary outcome measures

**Fig. 2:**
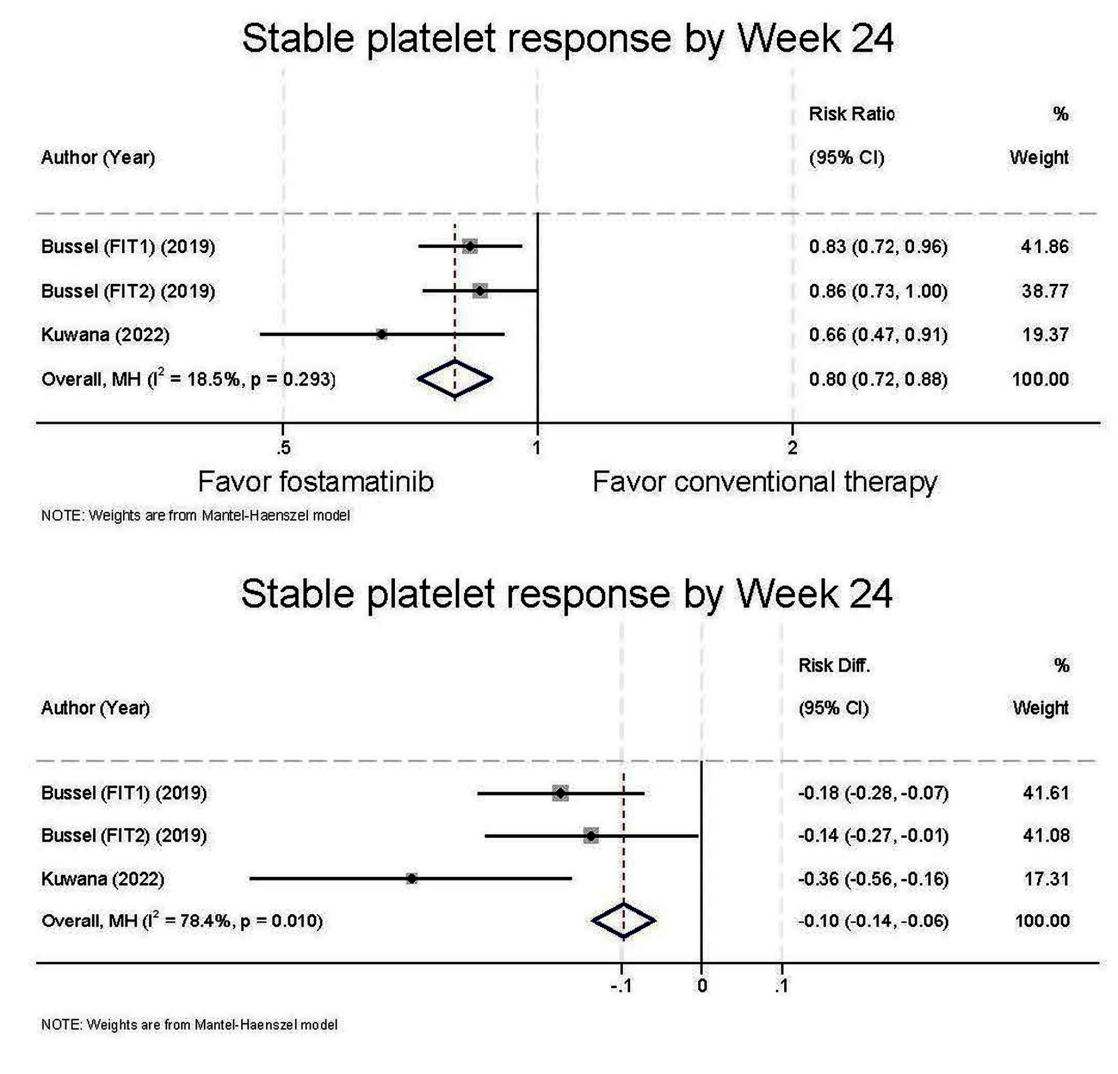
Comparison of platelet response by 24 weeks between fostamatinib therapy group and conventional group

The primary outcome in this study is efficacy measures by using the Objective Response Rate (ORR). The ORR for stable platelet response by week 24 was significantly higher in fostamatinib therapy group compared to conventional therapy alone, with an ORR of 0.80 (95%CI 0.72 to 0.88, I^2^ = 18.5%) (Fig. 2), favoring fostamatinib group. Heterogeneity analysis indicated a very low level of heterogeneity between studies (I^2^ = 18.5%). The pooled risk difference was − 0.10 (95%CI -0.14 to -0.06, I^2^ = 78.4%) and the Number Needed to Treat (NNT) was 10.

**Fig. 3:**
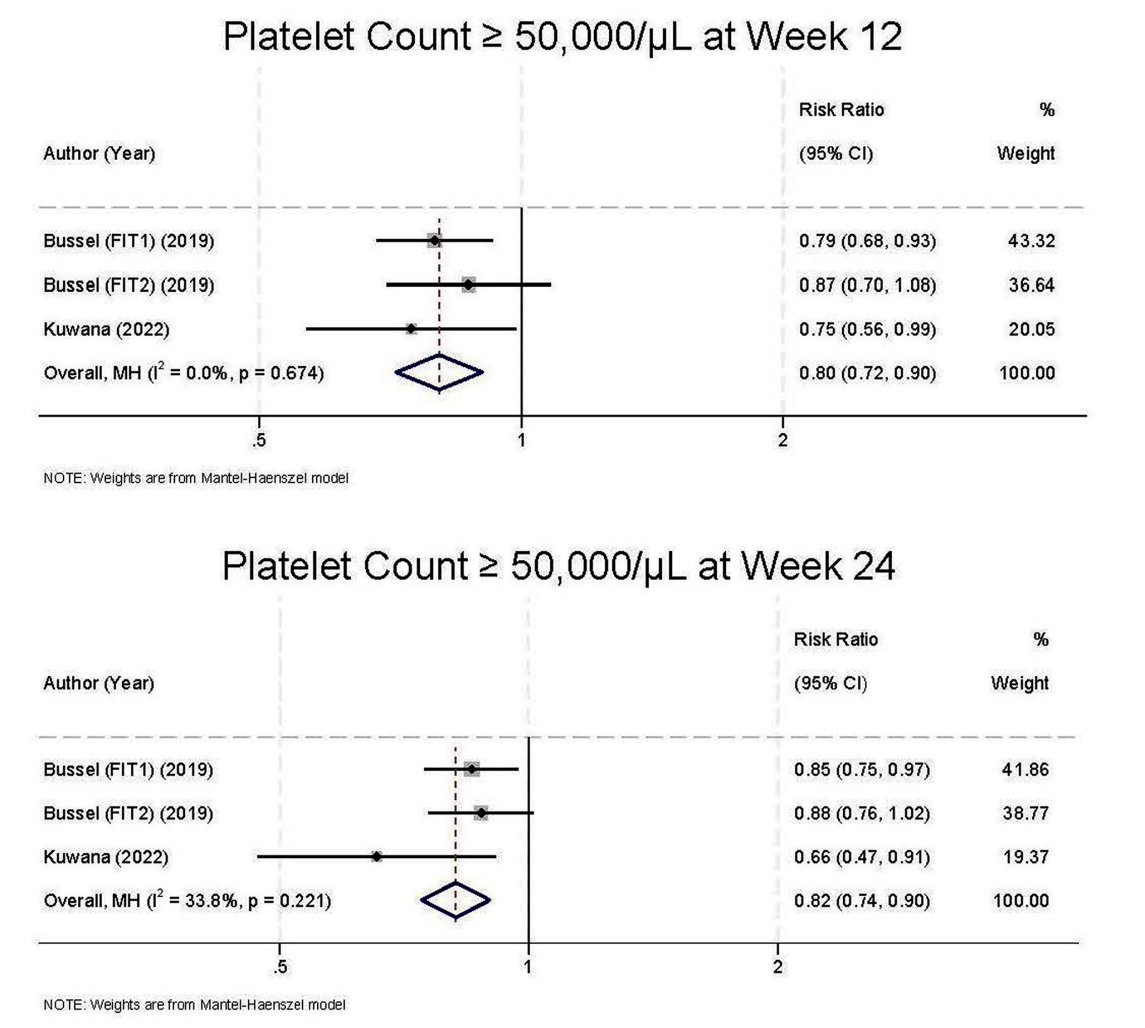
Comparison of outcome response rate at week 12 (**A**) and week 24 (**B**) between fostamatinib group and conventional group

The outcome of platelet Count ≥ 50,000/µL at week 12 ORR was 0.80 (95%CI 0.72 to 0.90, I^2^ = 0%) (Fig. 3). The outcome of platelet Count ≥ 50,000/µL at week 24 ORR was 0.82 (95%CI 0.72 to 0.90, I^2^ = 33.8%).

Among subjects with a baseline platelet count < 15,000/µL, achievement of a count ≥ 30,000/µL and at least 20,000/µL above baseline at week 12 ORR was 0.81 (95%CI 0.69 to 0.95, I^2^ = 0%), while achievement of a count ≥ 30,000/µL and at least 20,000/µL above baseline at week 24 ORR was 0.82 (95%CI 0.73 to 0.93, I^2^ = 0%) (Supplementary Figs. 2 and 3).

### Secondary outcome measure

**Fig. 4:**
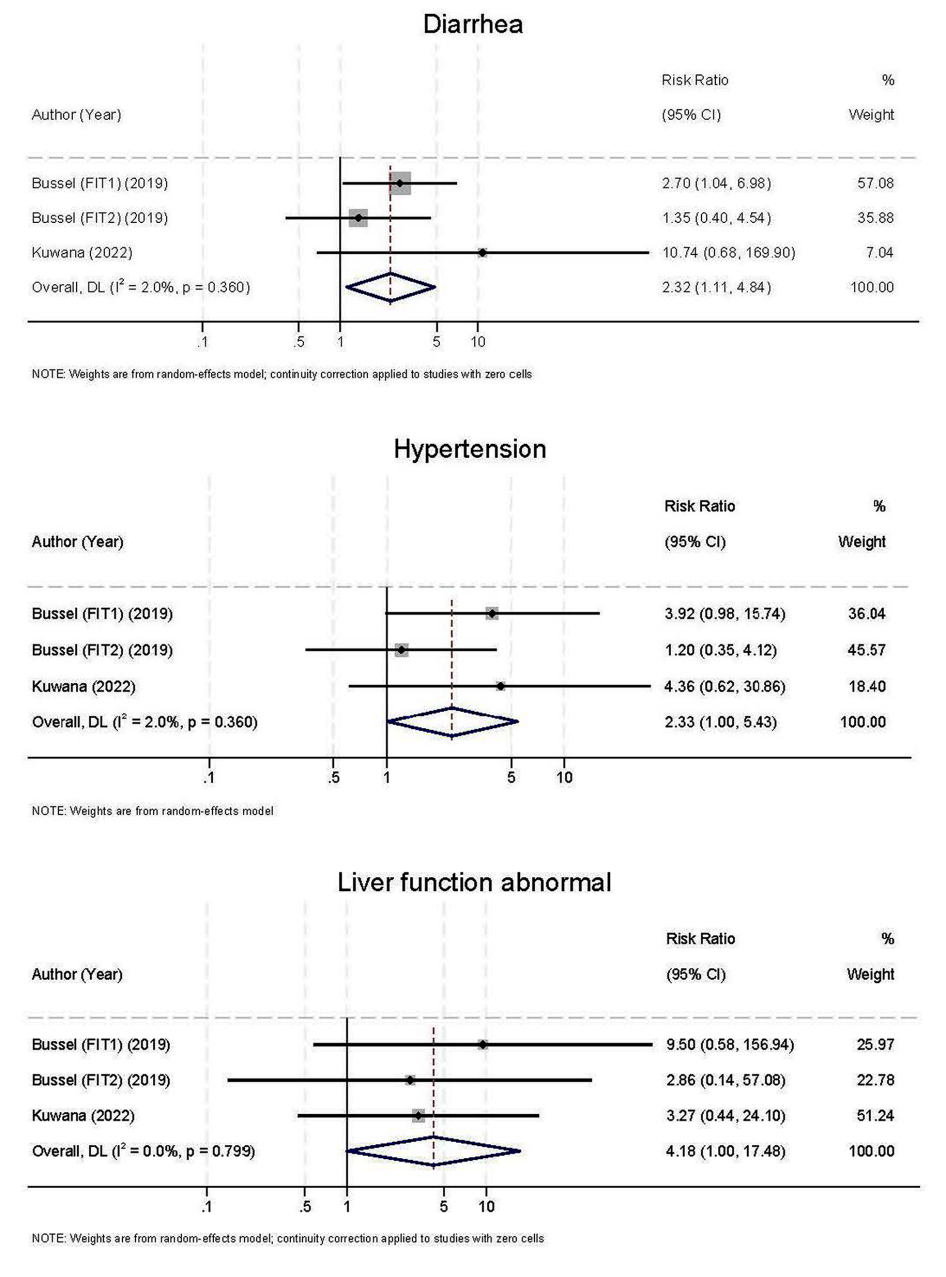
Side effects outcome between fostamatinib group and conventional group

Secondary outcome included adverse events. The result indicated that some AEs were significantly higher in fostamatinib group than conventional therapy such as diarrhea (RR = 2.32, RD = 0.23; 95%CI 0.02 to 0.44), hypertension (RR = 2.33, RD = 0.17; 95%CI 0.01 to 0.32), and liver function abnormality (RR = 4.18, RD = 0.12; 95%CI 0.02 to 0.22) (Fig. 4). Among side effects of any grade, nausea (RR = 1.77, RD = 0.08; 95%CI -0.09 to 0.26) and rash (RR = 2.28, RD = 0.06; 95%CI -0.01 to 0.12) were not statistically significant (Supplementary Figs. 4 and 5).

## Discussion

This meta-analysis, which examined the efficacy and side effects of fostamatinib, included three randomized controlled trials with 184 patients encompassing refractory ITP. Our primary outcome measures, specifically the Objective Response Rate (ORR), demonstrated significantly high values across all measured outcomes, such as stable platelet response by week 24. The consistent and favorable response to fostamatinib was evident across different time points. Given that this study was conducted in a refractory population, the Number Needed to Treat (NNT) of 10 appears to be a reasonable metric for clinical use. These findings reinforce the potential of fostamatinib as an effective therapeutic option for refractory ITP, aligning with previous studies that emphasized its role in improving platelet counts and achieving therapeutic goals.

In patients with ITP, head-to-head studies comparing treatments, mostly in persistent/chronic condition, are limited and a network meta-analysis approach is utilized to help clinicians decide between treatment options. We summarized the network meta-analysis in both newly diagnosed and chronic ITP (Tables 2 and 3, and 4). Treatment options for ITP vary in disease progression, effectiveness, and safety profiles. In primary ITP among children, a study in 2020 indicated that using 2 g/kg of IVIG results in a better response rate with fewer adverse reactions compared to other monotherapy options [16].

**Table 2:** Summarized the previous published network meta-analysis compared treatments in newly diagnosed ITP patients

Author	Population	Treatment	No. of study included	Result
Arai (2018) [17]	Newly diagnosed ITP	• PSL • Dex • RTX + Dex • IVIG +/- PSL • PSL (Low dose) • RTX + Dex + PSL • RTX + PSL • mPSL + PSL • rhTPO + Dex	21 RCTs (1898 pt)	• rhTPO + Dex and RTX + Dex are significantly better compared to PSL and Dex monotherapy • rhTPO + Dex and rhTPO + PSL improve early response compared to PSL, Dex, and RTX regimen • No difference in severe adverse event between groups
Acero-Garcés (2020) [16]	Primary ITP in children	• IVIG 2 g/kg • IVIG 0.8 g/kg • mPSL 30 mg/kg • mPSL 50 mg/kg • Placebo • PSL 2 mg/kg • PSL 4mgkg • Anti-D 50 µg/kg • Anti-D 75 µg/kg	12 RCTs (791 pt)	• IVIG 2 g/kg have better response rates when comparing to prednisolone 2 mg/kg and methylprednisolone 30 mg/kg • IVIG 2 g/kg had less adverse effect than Anti-D, methylprednisolone and IVIG 0.8 g/kg
Wang (2022) [18]	Newly diagnosed adult ITP	• TAC + Dex • RA + Dex • OSE + Dex • RTX + PSL • RTX + Dex • Dex • IVIG +/- PSL • PSL • PSL (Low dose) • Dex + rhTPO • mPSL +/- PSL	18 RCTs (1944 pt)	• Dex + rhTPO achieved the earliest response rate • RTX + PSL achieved the most 6 months sustained response • RTX + Dex showed the highest chances of grade 3 or higher adverse events by 15.3%

Abbreviation: anti-HP = anti-helicobacter pylori; Dex = Dexamethasone; IVIG = intravenous immunoglobulin; PSL = Prednisolone; mPSL = Methylprednisolone; RA = all-trans retinoic acid; RCTs = Randomized-controlled trials; RTX = Rituximab; TPO-RAs = Thrombopoietin Receptor Agonists; rhTPO = human recombinant thrombopoietin; TAC = tacrolimus; pt = participant

**Table 3:** Summarized the previous published network meta-analysis compared treatments in persistent/chronic ITP patients

Author	Population	Treatment	No. of study included	Result
Arai (2019) [19]	Persistent/Chronic ITP	• Romiplostim • Eltrombopag • Avatrombopag • RTX • rhTPO + RTX • Placebo	12 RCTs (1306 pt)	• TPO-RAs overall response superior to RTX or Placebo • No significant difference between two TPO-RAs • Bleeding event reduced with TPO-RAs • No increase in severe adverse event • Eltrombopag and Romiplostim can be considered as the first choice for patient with persistent or chronic ITP
Yang (2019) [14]	Previous treated ITP	• Eltrombopag • Romiplostim • RTX • Avatrombopag • Fostamatinib	13 RCTs (1202 pt)	• Romiplostim is the best in overall response followed by avatrombopag, eltrombopag, fostamatinib, and RTX • Avatrombopag is the best in early response (at 2 weeks) followed by romiplostim, eltrombopag, and RTX • No difference in severe adverse event between groups
Puavilai (2020) [20]	Persistent ITP	• Romiplostim • Eltrombopag • rhTPO + Danazol • rhTPO + cyclosporin • RTX • rhTPO + RTX	12 RCTs (1313 pt)	• Romiplostim and Eltrombopag are the best option relative to placebo • RTX may not be beneficial
Wojciech-owski (2021) [21]	Chronic ITP	• Avatrombopag • Eltrombopag • Fostamatinib • Placebo • Romiplostim	7 RCTs (470 pt)	• Avatrombopag was associated with statistically significant improvements in durable platelet response, reduction in use of concomitant ITP medication, and incidence of any bleeding events. • Avatrombopag had lower risk of bleeding when comparing to eltrombopag and romiplostim
Liu (2023) [22]	adult ITP	• Eltrombopag • Romiplostim • Avatrombopag • rhTPO • Hetrombopag	14 RCTs (1,360 pt)	• Avatrombopag and rhTPO showed better platelet response than eltrombopag • Avatrombopag showed highest platelet response with the least treatment-related adverse reaction • No significant differences in treatment related adverse reaction among eltrombopag, romiplostim and avatrombopag
Li (2023) [23]	Children and adults with persistent and chronic ITP	• Avatrombopag • Eltrombopag • Hetrombopag • Romiplostim • Placebo	15 RCTs (1563 pt)	• Adult patients treated with TPO-RAs had longer duration of platelet response, higher platelet response rate, lower use of rescue therapy, and lower incidence of bleeding events, and similar incidence of adverse events compared with placebo. Children showed similar outcomes to adults, with consistency across all parameters except for bleeding incidence. • Avatrombopag have more effective platelet responses rate than eltrombopag and hetrombopag in adult

Abbreviation: RCTs = Randomized-controlled trials; RTX = Rituximab; TPO-RAs = Thrombopoietin Receptor Agonists; rhTPO = human recombinant thrombopoietin; pt = participant

**Table 4:** Summarized the previous published network meta-analysis compared treatments in newly diagnosed and persistent ITP patients

Author	Population	Treatment	No. of study included	Result
Zhou (2022) [24]	Newly diagnosed and persistent ITP	• Placebo • Dex • Eltrombopag • PSL • Romiplostim • RTX • RTX + rhTPO • Immunosuppressant • IVIG • Fostamatinib • Eltrombopag + RTX • Efgartigimod • Dex + rhTPO • Dex + anti-HP • Dex + Oseltamivir • Danazol + steroid • Avatrombopag	19 RCTs (2615 pt)	• Eltrombopag + RTX is the best choice when start ITP treatment • Eltrombopag + RTX, avatrombopag, Dex + anti-HP, and Dex + rhTPO overall response better than placebo, alone Dex, PSL, and immunosuppressant • Fostamatinib cannot demonstrate higher efficacy than placebo

Abbreviation: anti-HP = anti-helicobacter pylori; Dex = Dexamethasone; IVIG = intravenous immunoglobulin; PSL = Prednisolone; RCTs = Randomized-controlled trials; RTX = Rituximab; rhTPO = human recombinant thrombopoietin; pt = participant

For newly diagnosed ITP, combination therapies have shown significantly better outcomes than prednisolone or dexamethasone monotherapy in terms of overall and sustained response. However, 15.3% of combination therapies between rituximab and dexamethasone reported grade 3 or higher adverse events [18]. Therefore, alternative combination therapies such as dexamethasone plus human recombinant thrombopoietin (rhTPO), rituximab plus prednisolone, or eltrombopag plus rituximab, as well as rhTPO plus prednisolone, are suggested due to their significant early response and favorable clinical outcomes with a lower chance of adverse reactions [17, 18, 24].

For persistent or chronic ITP, previous studies suggested that thrombopoietin receptor agonists (TPO-RAs) demonstrate superior clinical responses, including longer duration of platelet response, higher overall response rates, lower rescue therapy use, and fewer bleeding events compared to other treatment options. However, avatrombopag has shown a better platelet response rate than TPO-RAs with lower bleeding risk and no significant differences in treatment-related adverse reactions when compared to eltrombopag and romiplostim. Hence, it is recommended as an alternative treatment [19–23].

In the utilization of fostamatinib, previous studies are consistent with our findings, demonstrating a statistically significant increase in durable platelet response and superiority when compared to rituximab [14]. Additionally, it has shown a lower incidence of bleeding and demonstrated high cumulative probabilities of being the best treatment [21]. However, a meta-analysis in 2022, which included 19 drug therapies, contrasted that fostamatinib could not demonstrate higher efficacy than placebo. This disparity might be due to the very high heterogeneity of the studies, which included data from both newly diagnosed and chronic ITP patient, as well as the multitude of interventions studied, diluting the effect of fostamatinib [24].

There is no question regarding fostamatinib’s efficacy in chronic ITP patients. However, the question arises whether it can be used as a second-line treatment or beyond. Post hoc analysis data in 2020 suggested that fostamatinib was more effective as a second-line (78%) than third-or-later-line (48%) therapy for ITP [25]. Nevertheless, it’s crucial to note that fostamatinib exhibits a slower response compared to TPO-RA treatments, which often produce an “early response” effect within 2 weeks. This slower response might prompt clinicians, in severe cases, to consider other treatment options without waiting for fostamatinib’s response.

In alignment with prior research, our analysis underscores the challenges in managing ITP, particularly the variable response to conventional therapies and the potential for adverse effects. By pooling data from previous randomized controlled trials, we enhance the power to detect differences in outcomes and provide a more precise estimate of treatment effects. We noted that our study has very low heterogeneity between studies (I^2^ = 18.5%).The results of this meta-analysis have important implications for the management of refractory ITP. Despite some significant adverse events, for instance, diarrhea, hypertension, and liver function abnormal, the severity was less than grade 3. These mild side effects are consistent with the real-world data reported in 2023 and 2024 [26, 27]. However, physicians should be cautious in patients who have pre-existing conditions such as cirrhosis, hypertension, or gastrointestinal disease.

Although our study revealed promising data, there are some limitations due to the limited number of studies and participants included in this meta-analysis. Future research should focus on evaluating the long-term efficacy and safety of fostamatinib, as well as exploring potential strategies to mitigate the identified adverse events.

## Conclusion

Our meta-analysis underscores the potential of fostamatinib as a promising therapeutic option for adults with refractory immune thrombocytopenia (ITP). Fostamatinib, when combined with conventional therapy, demonstrated a significantly higher Objective Response Rate (ORR) in achieving stable platelet responses by week 24 and attaining desired platelet counts at both week 12 and 24 compared to conventional therapy alone. The consistent and favorable response observed across various time points emphasizes the drug’s efficacy in this challenging patient population.

Despite the promising efficacy, fostamatinib was associated with certain adverse events, including diarrhea, hypertension, and liver function abnormalities. However, the severity of these adverse events was generally mild (grade < 3), aligning with real-world data from recent years. Hence, while fostamatinib offers potential benefits, clinicians should be vigilant, particularly when considering patients with pre-existing conditions such as cirrhosis, hypertension, or gastrointestinal disease.

This meta-analysis provides valuable insights into the efficacy and safety profile of fostamatinib in refractory ITP and reinforces its potential as a viable treatment option. Nevertheless, the limited number of studies and participants in our analysis highlight the need for more extensive research to further validate these findings and explore strategies to minimize the identified adverse events. Future studies should also focus on assessing the long-term efficacy, safety, and optimal dosing strategies of fostamatinib to guide its appropriate use in the management of refractory ITP.

### Registration

This study was registered in the International Prospective Register of Systematic Reviews (PROSPERO) web registry. The registration number for this review is CRD42024528563.

### Electronic supplementary material

Below is the link to the electronic supplementary material.

Supplementary Material 1

## Data Availability

No datasets were generated or analysed during the current study.
