# Supplementary material for: Efficacy and safety of fostamatinib in refractory immune thrombocytopenia: a meta-analysis from randomized controlled trials
Source: Ann Hematol. 2024 Jun 10;103(9):3357–68. doi: 10.1007/s00277-024-05824-7 (PMC11358303; doi:10.1007/s00277-024-05824-7)
Supplement: Supplementary file 1 — Supplementary Material 1 [file 277_2024_5824_MOESM1_ESM.pdf]

Search queries

- **MEDLINE search strategy (via PubMed)**
  (“Immune thrombocytop*”[All Fields] OR “ITP”[All Fields]) AND ("fostamatinib"[All Fields] OR fostamatinib[Text Word] OR “R788”[Text Word]) NOT (animals[mh] NOT humans[mh])

- **Scopus search strategy**
  TITLE-ABS-KEY (“immune thrombocytop*”) OR TITLE-ABS-KEY (“ITP”)
  AND
  TITLE-ABS-KEY (“Fostamatinib”) OR TITLE-ABS-KEY (“R788”)

- **Embase search strategy**
  “Fostamatinib' AND (“immune thrombocytopenia” OR “itp”)

- **Clinicaltrial.gov search strategy**
  Condition or disease: “immune thrombocytopenia” OR “ITP”
  Intervention/Treatment: “Fostamatinib”

Item in data extraction form

GENERAL INFORMATIONS	• Study ID/Year
STUDY CHARACTERISTICS	• Design<br>• Country<br>• Randomization<br>• No. arm<br>• No. patient randomized (each arm)
PATIENTS CHARACTERISTICS	• No. of each gender (male/female)<br>• Age (year; median/min/max)<br>• Ethnicity<br>• Prior treatment history<br>• Platelet count at diagnosis<br>• Bleeding score at diagnosis<br>• Other complications
COMPONENT OF THE INTERVENTION	• Dosage, duration, interval, total amount, tapering<br>• Additional treatment (type, dosage, interval)
OUTCOMES	• Stable platelet response (n; by Week 24)<br>• Platelet response (n; at Week 12)<br>• Platelet response (n; at Week 24)<br>• Frequency and Severity of Bleeding According to the ITP Bleeding Score (IBLS)<br>• Frequency and Severity of Bleeding According to the World Health Organization (WHO) Bleeding Scale<br>• Types of side effects<br>• No. of patient (each side effects)
RISK OF BIAS	• Randomisation process<br>• Allocation concealment<br>• Deviations from the intended interventions<br>• Blinding of participants and personnel<br>• Blinding of outcome assessment<br>• Missing outcome data (efficacy / safety)<br>• Selection of the reported result

Risk of Bias assessment

The RoB assessment covers five domains, each of which was evaluated based on the criteria outlined in the tool.

1.Randomization Process: All studies provided detailed information about the allocation sequences, which were stratified random. Two studies balanced patients with prior splenectomy and the degree of thrombocytopenia, while one study stratified by baseline platelet count (< or ≥15 000/µl). Conflict arose in the allocation concealment part; while one author (J.J) rated all studies as “No information,” the other authors (S.T. and N.B) pointed that in the context of a large trial run by an experienced clinical trials unit, responding with "Probably yes" rather than "No information" is more reasonable. Our judgment differed from a previous meta-analysis, which rated it as some concern due to no information. No baseline differences between treatment arms were observed. We rated low risk in this domain.

2.Deviation from Intended Interventions: We assessed this domain as low risk, as all studies employed double-blind designs, ensuring that participants and physicians were unaware of their assigned interventions during the trial. Additionally, the primary outcome measure, platelet count evaluation, was conducted consistently across all studies, aligning with the intended intervention protocols.

3.Missing Outcome Data: Our assessment of this domain resulted in a low-risk rating, as all data relevant to the outcomes of interest were available for analysis. There were no instances of missing outcome data reported in the included studies, minimizing the risk of bias associated with incomplete outcome reporting.

4.Measurement of the Outcome (detection bias): We rated this domain as low risk because the measurement of outcomes was consistent and comparable between treatment groups across all included studies. The outcome measures were well-defined and standardized, facilitating accurate assessment and interpretation of treatment effects.

5.Selection of the Reported Result (reporting bias): Our evaluation of this domain yielded a low-risk rating as the data analysis was conducted according to pre-specified analysis plans established before the randomization process. This approach minimizes the risk of selective reporting bias and enhances the transparency and reliability of the reported results.

Intention-to-treat

Unique ID	Study ID	Experimental	Comparator	D1	D2	D3	D4	D5	Overall
1	NCT02076399	Fostamatinib	Conventional therapy	+	+	+	+	+	+
2	NCT02076412	Fostamatinib	Conventional therapy	+	+	+	+	+	+
3	Kuwana et al.	Fostamatinib	Conventional therapy	+	+	+	+	+	+

+	Low risk
!	Some concerns
-	High risk

D1	Randomisation process
D2	Deviations from the intended interventions
D3	Missing outcome data
D4	Measurement of the outcome
D5	Selection of the reported result

Supplementary Figure 1

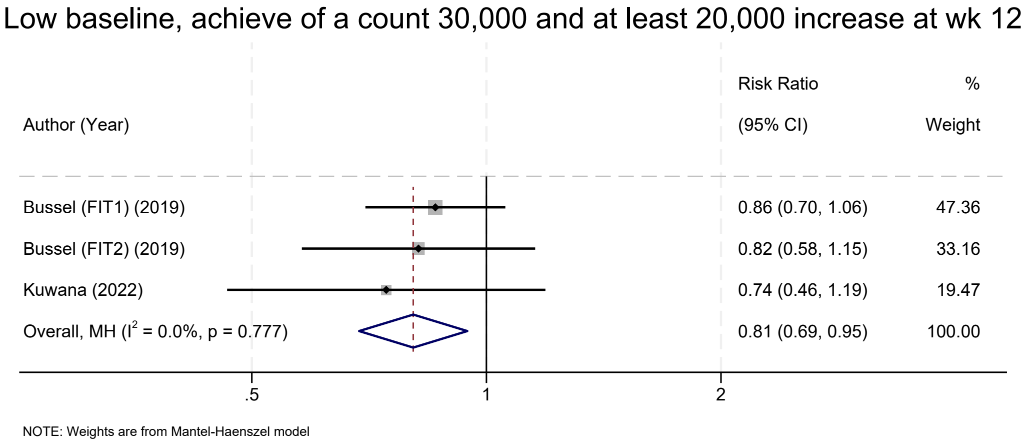

Supplementary Figure 2

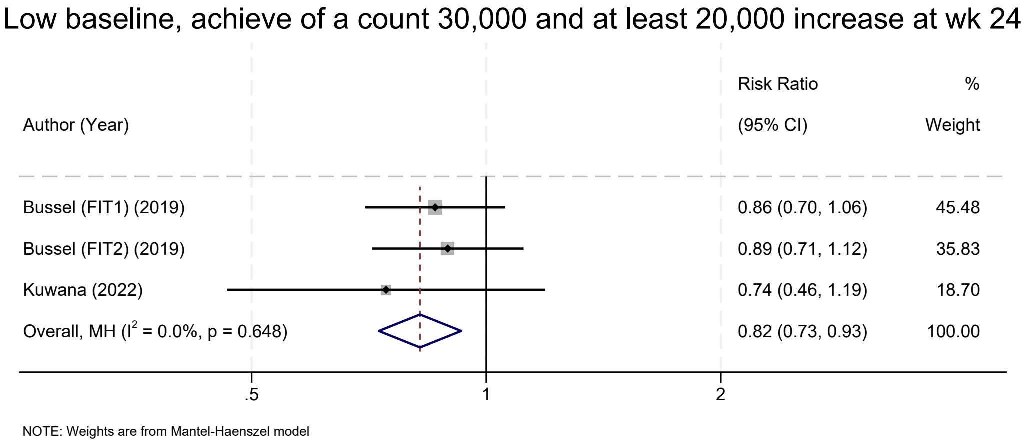

Supplementary Figure 3

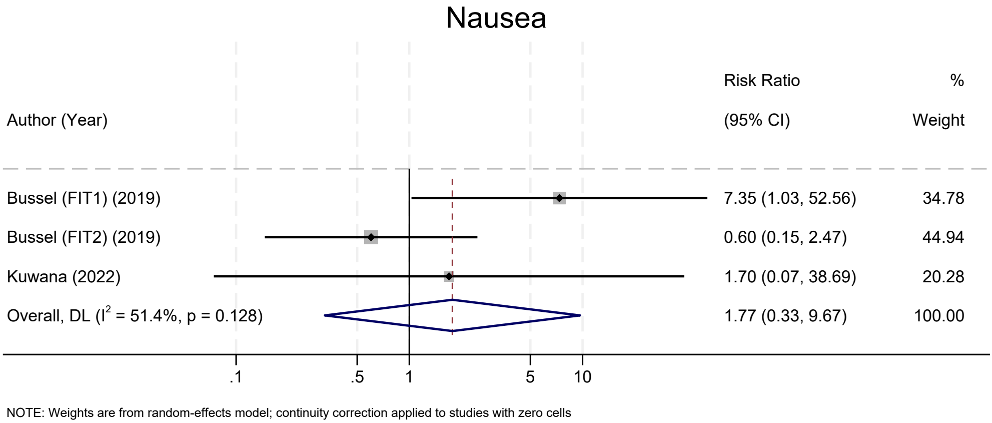

Supplementary Figure 4

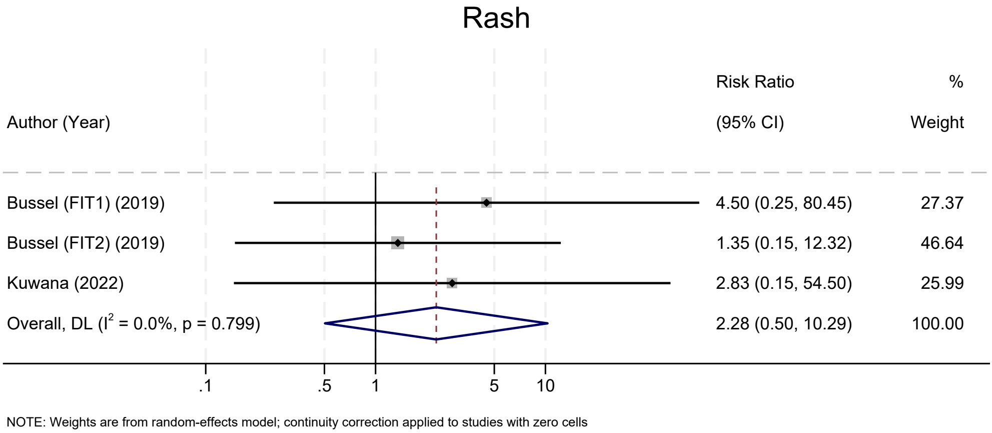

Supplementary Figure 5

PRISMA 2020 for Abstracts Checklist

Section and Topic	Item #	Checklist item	Reported (Yes/No)
**TITLE**			
Title	1	Identify the report as a systematic review.	Yes
**BACKGROUND**			
Objectives	2	Provide an explicit statement of the main objective(s) or question(s) the review addresses.	Yes
**METHODS**			
Eligibility criteria	3	Specify the inclusion and exclusion criteria for the review.	Yes
Information sources	4	Specify the information sources (e.g. databases, registers) used to identify studies and the date when each was last searched.	Yes
Risk of bias	5	Specify the methods used to assess risk of bias in the included studies.	Yes
Synthesis of results	6	Specify the methods used to present and synthesise results.	Yes
**RESULTS**			
Included studies	7	Give the total number of included studies and participants and summarise relevant characteristics of studies.	Yes
Synthesis of results	8	Present results for main outcomes, preferably indicating the number of included studies and participants for each. If meta-analysis was done, report the summary estimate and confidence/credible interval. If comparing groups, indicate the direction of the effect (i.e. which group is favoured).	Yes
**DISCUSSION**			
Limitations of evidence	9	Provide a brief summary of the limitations of the evidence included in the review (e.g. study risk of bias, inconsistency and imprecision).	Yes
Interpretation	10	Provide a general interpretation of the results and important implications.	Yes
**OTHER**			
Funding	11	Specify the primary source of funding for the review.	No
Registration	12	Provide the register name and registration number.	No

From: Page MJ, McKenzie JE, Bossuyt PM, Boutron I, Hoffmann TC, Mulrow CD, et al. The PRISMA 2020 statement: an updated guideline for reporting systematic reviews. BMJ 2021;372:n71. doi: 10.1136/bmj.n71

For more information, visit: http://www.prisma-statement.org/

PRISMA 2020 Checklist

Section and Topic	Item #	Checklist item	Location where item is reported
**TITLE**			
Title	1	Identify the report as a systematic review.	Line 7
**ABSTRACT**			
Abstract	2	See the PRISMA 2020 for Abstracts checklist.	
**INTRODUCTION**			
Rationale	3	Describe the rationale for the review in the context of existing knowledge.	Line 76
Objectives	4	Provide an explicit statement of the objective(s) or question(s) the review addresses.	Line 79
**METHODS**			
Eligibility criteria	5	Specify the inclusion and exclusion criteria for the review and how studies were grouped for the syntheses.	Line 108
Information sources	6	Specify all databases, registers, websites, organisations, reference lists and other sources searched or consulted to identify studies. Specify the date when each source was last searched or consulted.	Line 100
Search strategy	7	Present the full search strategies for all databases, registers and websites, including any filters and limits used.	Supplementary
Selection process	8	Specify the methods used to decide whether a study met the inclusion criteria of the review, including how many reviewers screened each record and each report retrieved, whether they worked independently, and if applicable, details of automation tools used in the process.	Line 114
Data collection process	9	Specify the methods used to collect data from reports, including how many reviewers collected data from each report, whether they worked independently, any processes for obtaining or confirming data from study investigators, and if applicable, details of automation tools used in the process.	Line 117
Data items	10a	List and define all outcomes for which data were sought. Specify whether all results that were compatible with each outcome domain in each study were sought (e.g. for all measures, time points, analyses), and if not, the methods used to decide which results to collect.	Supplementary
	10b	List and define all other variables for which data were sought (e.g. participant and intervention characteristics, funding sources). Describe any assumptions made about any missing or unclear information.	Supplementary
Study risk of bias assessment	11	Specify the methods used to assess risk of bias in the included studies, including details of the tool(s) used, how many reviewers assessed each study and whether they worked independently, and if applicable, details of automation tools used in the process.	Line 125
Effect measures	12	Specify for each outcome the effect measure(s) (e.g. risk ratio, mean difference) used in the synthesis or presentation of results.	Line 130
Synthesis methods	13a	Describe the processes used to decide which studies were eligible for each synthesis (e.g. tabulating the study intervention characteristics and comparing against the planned groups for each synthesis (item #5)).	n/a
	13b	Describe any methods required to prepare the data for presentation or synthesis, such as handling of missing summary statistics, or data conversions.	n/a
	13c	Describe any methods used to tabulate or visually display results of individual studies and syntheses.	140
	13d	Describe any methods used to synthesize results and provide a rationale for the choice(s). If meta-analysis was performed, describe the model(s), method(s) to identify the presence and extent of statistical heterogeneity, and software package(s) used.	Line 129
	13e	Describe any methods used to explore possible causes of heterogeneity among study results (e.g. subgroup analysis, meta-regression).	n/a
	13f	Describe any sensitivity analyses conducted to assess robustness of the synthesized results.	n/a
Reporting bias assessment	14	Describe any methods used to assess risk of bias due to missing results in a synthesis (arising from reporting biases).	Supplementary
Certainty assessment	15	Describe any methods used to assess certainty (or confidence) in the body of evidence for an outcome.	Supplementary

PRISMA 2020 Checklist

Section and Topic	Item #	Checklist item	Location where item is reported
**RESULTS**			
Study selection	16a	Describe the results of the search and selection process, from the number of records identified in the search to the number of studies included in the review, ideally using a flow diagram.	Line 135
	16b	Cite studies that might appear to meet the inclusion criteria, but which were excluded, and explain why they were excluded.	n/a
Study characteristics	17	Cite each included study and present its characteristics.	Table1
Risk of bias in studies	18	Present assessments of risk of bias for each included study.	Supplementary
Results of individual studies	19	For all outcomes, present, for each study: (a) summary statistics for each group (where appropriate) and (b) an effect estimate and its precision (e.g. confidence/credible interval), ideally using structured tables or plots.	Figure 2, 3, 4
Results of syntheses	20a	For each synthesis, briefly summarise the characteristics and risk of bias among contributing studies.	n/a
	20b	Present results of all statistical syntheses conducted. If meta-analysis was done, present for each the summary estimate and its precision (e.g. confidence/credible interval) and measures of statistical heterogeneity. If comparing groups, describe the direction of the effect.	Line 183-200
	20c	Present results of all investigations of possible causes of heterogeneity among study results.	n/a
	20d	Present results of all sensitivity analyses conducted to assess the robustness of the synthesized results.	n/a
Reporting biases	21	Present assessments of risk of bias due to missing results (arising from reporting biases) for each synthesis assessed.	n/a
Certainty of evidence	22	Present assessments of certainty (or confidence) in the body of evidence for each outcome assessed.	Line 184, 186
**DISCUSSION**			
Discussion	23a	Provide a general interpretation of the results in the context of other evidence.	Line 217
	23b	Discuss any limitations of the evidence included in the review.	Line 263
	23c	Discuss any limitations of the review processes used.	n/a
	23d	Discuss implications of the results for practice, policy, and future research.	Line 256
**OTHER INFORMATION**			
Registration and protocol	24a	Provide registration information for the review, including register name and registration number, or state that the review was not registered.	Line 289
	24b	Indicate where the review protocol can be accessed, or state that a protocol was not prepared.	n/a
	24c	Describe and explain any amendments to information provided at registration or in the protocol.	n/a
Support	25	Describe sources of financial or non-financial support for the review, and the role of the funders or sponsors in the review.	Line 293
Competing interests	26	Declare any competing interests of review authors.	Line 292
Availability of data, code and other materials	27	Report which of the following are publicly available and where they can be found: template data collection forms; data extracted from included studies; data used for all analyses; analytic code; any other materials used in the review.	Supplementary

From: Page MJ, McKenzie JE, Bossuyt PM, Boutron I, Hoffmann TC, Mulrow CD, et al. The PRISMA 2020 statement: an updated guideline for reporting systematic reviews. BMJ 2021;372:n71. doi: 10.1136/bmj.n71

For more information, visit: http://www.prisma-statement.org/
